# Helminth-induced CD19^+^CD23^hi^ B cells modulate experimental allergic and autoimmune inflammation

**DOI:** 10.1002/eji.200939721

**Published:** 2010-03-19

**Authors:** Mark S Wilson, Matthew D Taylor, Mary T O'Gorman, Adam Balic, Tom A Barr, Kara Filbey, Stephen M Anderton, Rick M Maizels

**Affiliations:** Centre for Immunity, Infection and Evolution, and Institute of Immunology and Infection Research, University of EdinburghEdinburgh, UK

**Keywords:** Allergology, Autoimmunity, B cells, Immune regulation, Parasitology

## Abstract

Numerous population studies and experimental models suggest that helminth infections can ameliorate immuno-inflammatory disorders such as asthma and autoimmunity. Immunosuppressive cell populations associated with helminth infections include Treg and alternatively-activated macrophages. In previous studies, we showed that both CD4^+^CD25^+^ Treg, and CD4^–^ MLN cells from *Heligmosomoides polygyus*-infected C57BL/6 mice were able to transfer protection against allergic airway inflammation to sensitized but uninfected animals. We now show that CD4^–^CD19^+^ MLN B cells from infected, but not naïve, mice are able to transfer a down-modulatory effect on allergy, significantly suppressing airway eosinophilia, IL-5 secretion and pathology following allergen challenge. We further demonstrate that the same cell population can alleviate autoimmune-mediated inflammatory events in the CNS, when transferred to uninfected mice undergoing myelin oligodendrocyte glycoprotein_(p35–55)_-induced EAE. In both allergic and autoimmune models, reduction of disease was achieved with B cells from helminth-infected IL-10^−/−^ donors, indicating that donor cell-derived IL-10 is not required. Phenotypically, MLN B cells from helminth-infected mice expressed uniformly high levels of CD23, with follicular (B2) cell surface markers. These data expand previous observations and highlight the broad regulatory environment that develops during helminth infections that can abate diverse inflammatory disorders *in vivo*.

## Introduction

The question of if, and how, infection may modulate the severity of allergic and autoimmune pathologies is steadily gaining prominence, reflecting the continuing rise in prevalence of immunopathologies, and the evidence for an inverse correlation between viral, bacterial and parasitic infections with rising asthma, multiple sclerosis and Crohn's disease [1–6].

Until recently, the Th1-Th2 dichotomy was invoked to explain the regulation of immunopathologic disease. Thus, reduced Th1-associated autoimmune pathologies were observed following Th2-inducing helminth infections [7–9], while, Th2-driven allergic reactions are reduced following infection with Th1-inducing pathogens [10–12]. However, Th2-inducing human helminth infections are often protective against Th2-mediated responses to allergen provocation [13–15], while autoimmunity can be blocked by some bacterial pathogens [16].

These studies have led beyond the Th1/Th2 paradigm to a recognition that a balance between immunoregulatory and effector mechanisms is required to keep allergic or autoimmune propensities in check [17–21]. Furthermore, we, and others, have shown that parasitic infections can expand Treg cell populations [22–27], including the demonstration that adoptively transferred *H. polygyrus*-induced CD4^+^CD25^+^ Treg cells suppress inflammation following airway allergen challenge [23].

Although the Treg (CD4^+^CD25^+^Foxp3^+^) is a central player in the immunoregulatory network, in the contexts of both airway allergy [28, 29] and autoimmune disease [21, 30], this is not the only cell population capable of down-regulating immune responsiveness. Suppressive, alternatively activated macrophages are able to directly block T-cell proliferation, and are selectively enhanced by helminth infection [31–34]. Recently evidence has emerged for B cells expressing regulatory function to control immune pathologies [35–39], and in the context of helminth infections in mice [40–43] as well as humans [44].

We accordingly set out to test whether B cells, generated during chronic *H. polygyrus* infection, were capable of regulating allergen-induced airway inflammation (AAI), as might be predicted from our earlier finding that CD4^−^ MLN cells (MLNC) from infected C57BL/6 mice could transfer protection to uninfected, allergen-sensitized animals [23]. As shown here, CD4^−^CD19^+^ B cells from chronically infected mice can indeed suppress airway inflammation in uninfected recipients, reducing bronchoalveolar IL-5, eosinophilia and airway pathology. Furthermore, the same population of CD4^−^CD19^+^ B cells from infected donors can, on transfer, significantly reduce the severity of disease during EAE in uninfected recipients. We demonstrate that in neither setting is IL-10 required from the donor B-cell population to protect against pathogenic inflammation of these different aetiologies *in vivo*.

## Results

### Both CD4^+^ and CD4^−^ MLNC from *H. polygyrus*-infected C57BL/6 mice can mediate suppression

*H. polygyrus* is a natural enteric-dwelling nematode parasite which establishes a chronic infection in many strains of mice [45]. We have previously shown that *H. polygyrus*-infected mice display significantly reduced airway inflammatory responses to OVA or Der p1 allergen provocation in BALB/c and C57BL/6 mice, respectively [23]. Notably, in this system suppression of the allergic outcome was not associated with a shift from Th2 to Th1 responsiveness. In fact, as demonstrated by adoptive transfer, MLN CD4^+^CD25^+^ Treg cells from infected animals of both strains were able to transfer protection from airway inflammation in uninfected recipients [23].

**Figure 1:**
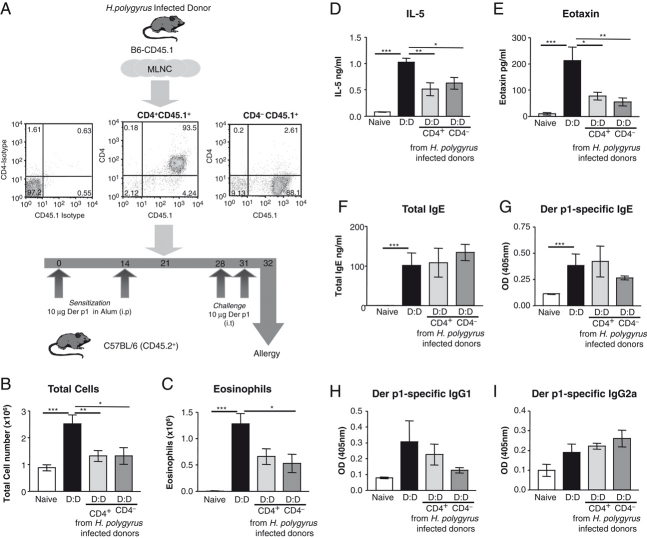
CD4^+^ and CD4^−^ cells from helminth-infected donors can suppress Der p1-induced airway inflammation. 4×10^6^ purified CD4^+^ and CD4^−^ MLNC from 28 day *H.polygyrus*-infected B6-CD45.1 mice were injected i.v. into C57BL/6 (CD45.2^+^) recipients that had received Der p1 immunizations 7 and 21 days earlier (day 0 and 14 in (A), respectively). Seven and 10 days after transfer (days 28 and 31, respectively), the mice were challenged with Derp1 and measurements made 1 day later (day 32). D:D denotes mice receiving Der p1 immunizations and challenge. Naïve indicates uninfected, non-immunised or challenged mice. (A) Schematic of the transfer protocol and flow cytometry plots showing the purity of the MACS-sorted CD4^+^ and CD4^−^ MLNC prior to transfer; (B) total cell numbers, (C) eosinophil numbers, (D) IL-5 levels and (E) eotaxin levels in BAL, (F) total IgE, (G) Der p 1-specific IgE, (H) Der p 1-specific IgG1 and (I) Der p 1-specific IgG2a in serum. Data are means±SE from five individual mice *per* group. Statistical analysis by the Mann–Whitney test: ^*^p<0.04; ^**^p<0.02; ^***^p<0.004.

An intriguing feature of our earlier study [23] was that, in the C57BL/6 strain, CD4^−^ MLNC were also able to transfer significant protection against airway allergy. To investigate this further, we first extended studies in the transfer model (Fig. 1A) to compare airway cell infiltration and inflammatory cytokine production in allergic mice receiving CD4^+^ (>93% pure) or CD4^−^ (>97%) MLNC from *H. polygyrus*-infected mice, employing an allotypic difference in CD45 (Ly5) to permit subsequent tracking of transferred cells [46]. Allergen-sensitized C57BL/6 (CD45.2/Ly5.2^+^) mice received 4×10^6^ CD4^+^ or CD4^−^ MLNC from infected congenic B6-CD45.1 donors i.v. 7 days before the first of two (day 28 and day 31) intratracheal Der p1 airway challenges. Mice were assessed for airway inflammation on day 32.

Der p1 airway challenge of Der p1-sensitized mice incited a substantial cellular influx into the BALF with a significant proportion of eosinophils, characteristic of allergen-induced inflammation. As previously observed in the C57BL/6 system [23], CD4^+^ and CD4^−^ MLNC showed equivalent efficacy at reducing total airway cell infiltration (Fig. 1B) and eosinophilia (Fig. 1C) in recipient mice. Moreover, the two cell populations were broadly similar in reduction of IL-5 (Fig. 1D) and eotaxin (Fig. 1E) in the BALF, consistent with the attenuated degree of eosinophilia.

Because the transferred CD4^−^ population contains approximately 70% B cells, we also examined whether total and allergen-specific antibody responses were altered in recipient animals. Neither total nor Der p 1-specific IgE levels showed significant differences between control allergic mice and those receiving cell transfers (Fig. 1F and G), as found previously for BALB/c mice [23]. IgG isotype analysis did indicate a reduction in Th2-associated IgG1, and a small increment in Th1-linked IgG2a in the CD4^−^ MLNC recipients (Fig. 1H and I), but not at levels that achieved statistical significance.

**Figure 2:**
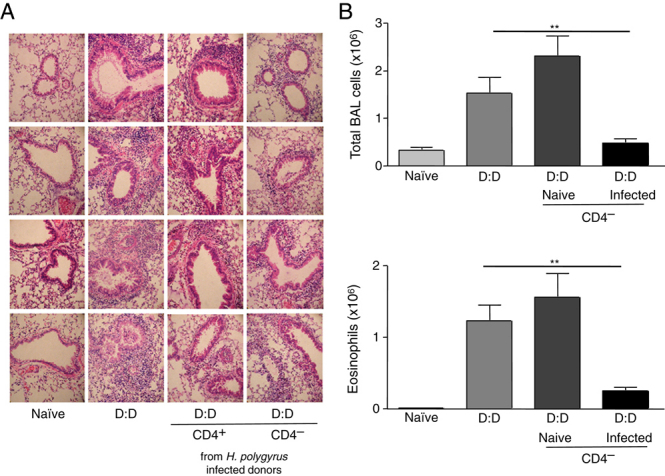
Reduced airway pathology and inflammation following adoptive transfer of CD4^−^ cells from helminth-infected donors. Experiments were performed as in Fig. 1A with transfer of purified CD4^+^ and CD4^−^ MLNC from 28 day *H.polygyrus*-infected mice. (A) On day 32 lung sections were stained with hematoxylin and eosin to measure cellular inflammation within the broncho-vascular bundles following airway challenge. D:D denotes mice receiving Der p1 immunizations and challenge. Four individual mice are shown for each group. (B) Experiments were performed as in Fig. 1A with transfer of CD4^−^ cells from naïve or 28 day *H.polygyrus*-infected mice. Airway inflammation was measured by total cell and eosinophil numbers in BAL. Statistical analysis by the Mann–Whitney test: ^**^p<0.02.

In addition to the suppression of airway eosinophilia and local IL-5 and eotaxin secretions, airway pathology was also attenuated. As expected, a dense layer of cells was observed surrounding the bronchioles and arteries of Der p1-sensitized mice following Der p1 challenge (Fig. 2A). The adoptive transfer of CD4^+^ or CD4^−^ cells from chronically infected donors substantially decreased peri-bronchial and peri-arterial inflammation following Der p1 challenge (Fig. 2A). In a further experiment it was confirmed that down-modulation of airway inflammation and pathology was affected only by CD4^−^ cells from infected, and not uninfected, mice (Fig. 2B).

### CD4^+^ and CD4^−^ cells from infected donors traffic to the airways

**Figure 3:**
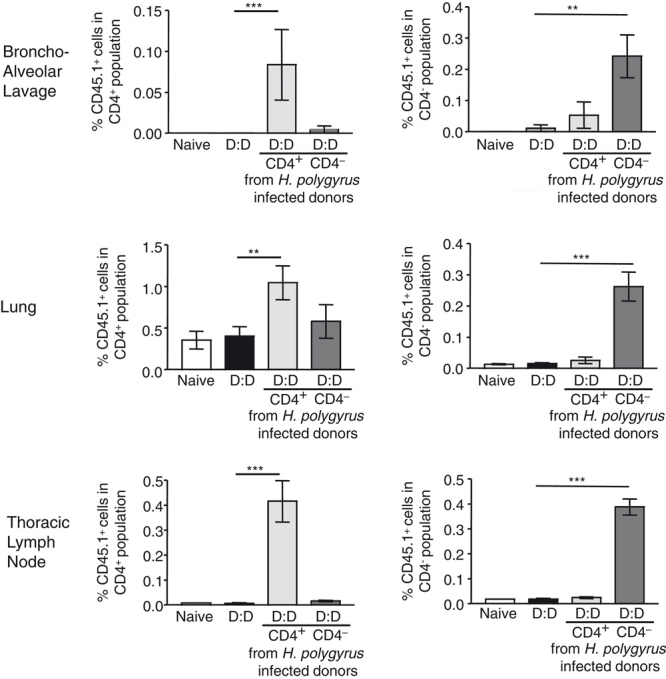
Donor cells traffic to allergen-exposed airways, lung interstitium and local draining LN. Experiments were performed as in Fig 1A by transfer of MLNC from 28 day *H.polygyrus*-infected B6-CD45.1 mice into C57BL/6 (CD45.2^+^) recipients, and the numbers of donor (CD45.1^+^) CD4^+^ and CD4^−^ cells in BAL, lung and thoracic LN determined. Data are means±SE from five individual mice *per* group. Statistically significant differences, assessed by the Mann–Whitney test; *p*<0.02; ^***^p<0.004.

The CD45 congenic marker permitted us to track the location of donor cells in recipient mice at the end-point of the experiment. At day 32, 11 days after the transfer of cells from infected donors, we found a significant accumulation of donor CD4^+^ and CD4^−^ cells in the airspace, lung tissue and draining thoracic LNs of Der p1-challenged mice (Fig. 3). These data suggest that both CD4^+^ and CD4^−^ populations migrate to the sites and local draining nodes responding to allergen provocation and suppress allergen-induced IL-5 and eotaxin secretion, reducing the recruitment of eosinophils.

### CD19^+^ B cells from infected WT and IL-10^−/−^ mice suppress allergen-induced airway eosinophilia

MLNC from chronically-infected mice consist of approximately 40% CD4^+^ T cells (Supporting Information Fig. 1A, lower left panel) and a similar proportion of CD19^+^ B Cells (Supporting Information. Fig. 1A, lower right panel). We therefore purified CD19^+^ B cells, representing the majority of the CD4^−^ population, from the MLN of naïve and chronically infected WT C57BL/6 mice for functional testing in the transfer model (Supporting Information Fig. 1B). Because of the prominent role of IL-10 in many B-cell regulatory functions [40, 41, 47] we also purified CD19^+^ B cells from chronically infected IL-10^−/−^ mice for assay in the same system.

**Figure 4:**
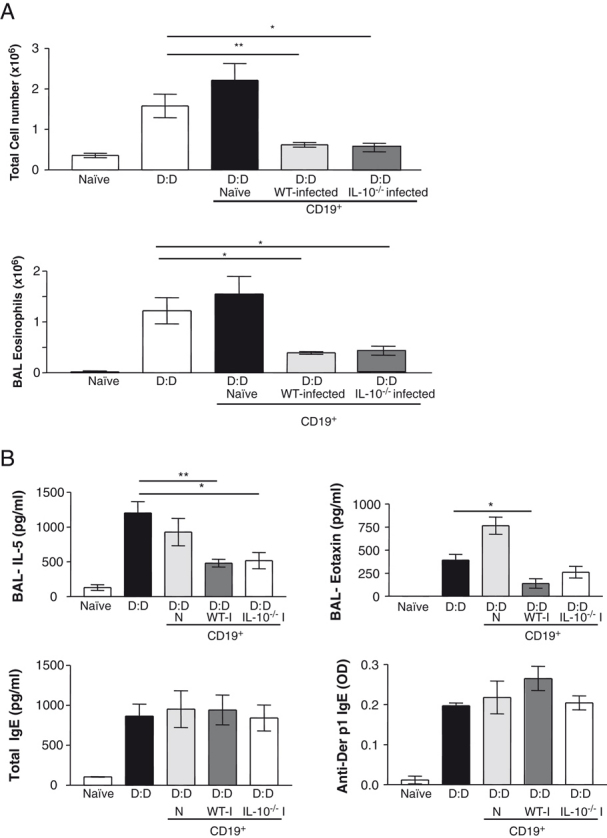
CD19^+^ cells inhibit allergen-induced airway inflammation, independent of donor IL-10. Experiments were performed as in Fig. 1A with the transfer of purified CD19^+^ cells from naïve, WT-infected and IL-10^−/−^-infected mice. (A) Total cell and eosinophil infiltration into the BALF. (B) IL-5 and eotaxin concentrations in BALF and total IgE and Der p 1-specific IgE concentrations in serum. Data are means±SE from five individual mice *per* group. Statistically significant differences, assessed by the Mann–Whitney test: ^*^p<0.04; ^**^p<0.02.

CD19^+^ B cells were transferred from the MLN of naïve or infected mice, into Der p1-sensitized recipients, 7 days before the first of two Der p1 airway challenges. Mice receiving CD19^+^ cells from chronically infected donors had significantly fewer cells infiltrating the airspaces following Der p1 challenge while CD19^+^ cells from uninfected donors (naïve) had no effect on airway infiltration (Fig. 4A). Similarly, airway eosinophilia was significantly ablated by the introduction of B cells from infected, but not naïve, donors. Importantly, the ability of B cells from infected mice to block allergic reactions was evident in both total cell numbers and eosinophilia irrespective of IL-10 competence.

Furthermore, secretions of IL-5 and eotaxin recovered from the airspaces were also significantly decreased following the transfer of B cells from chronically infected WT or IL-10-deficient donors, while transfer of naïve B cells had no effect (Fig. 4B). In contrast, B-cell transfers exerted no influence on the overall levels of serum IgE, or on allergen-specific IgE responses, as observed previously in recipients of total CD4^−^ cell populations, and neither total nor allergen-specific IgA were significantly altered in recipients of B cells from naïve or infected mice (data not shown).

### CD4^+^ and CD4^−^ cells from infected donors modulate the severity of MOG_(p35–55)_-induced EAE

**Figure 5:**
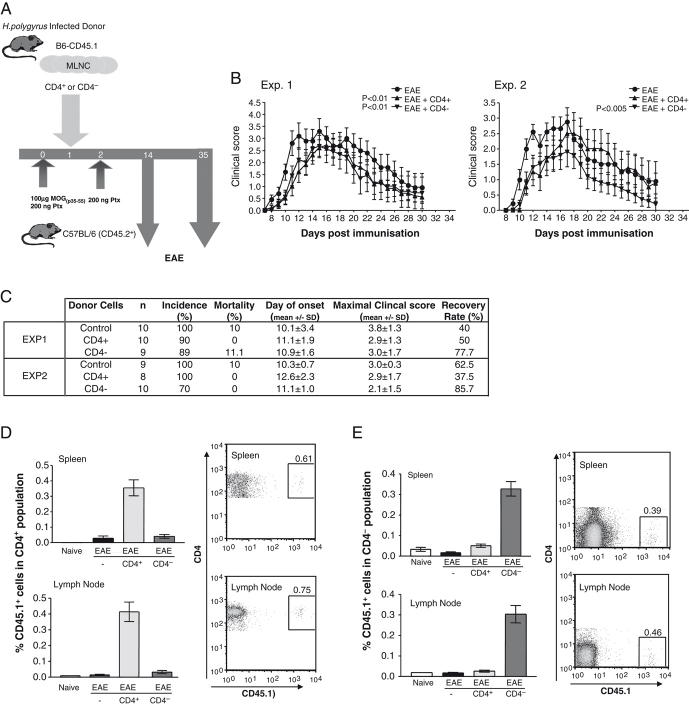
CD4^+^ and CD4^−^ cells from helminth-infected donors can also traffic to CNS-draining LN and suppress MOG_(p35–55)_-induced EAE. Transfer of 4×10^6^ CD4^+^ and CD4^−^ MLNC from day 28 *H.polygyrus*-infected B6-CD45.1 mice into C57BL/6 recipients that had been immunised with MOG_(p35–55)_ plus pertussis toxin (Ptx) 1 day before (day 0 in scheme). One day after transfer, Ptx was injected again; between 12 and 33 days later (days 14 and 35, respectively) the mice were assessed for the development of EAE. (A) Experimental design of transfer. (B) Effect of transferred CD4^+^ and CD4^−^ MLN cells from day 28-infected donors on MOG_(p35–55)_-induced EAE. (C) Summary of incidence, mortality and recovery from EAE of the experiments presented in (B). Numbers of (D) CD4^+^ and (E) CD4^−^ cells in the spleen and popliteal/inguinal LN. (D and E) Data in bar charts are means±SE from ten individual mice *per* group, with representative flow cytometry plots presented alongside.

The capacity of helminth-induced cell populations to regulate inflammatory disorders was further examined in an autoimmune setting, in which myelin oligodendrocyte glycoprotein (MOG) reactive T cells cause EAE. Using the CD45.1/2 adoptive transfer system, we first tested whether CD4^+^or CD4^−^ populations from the MLN of chronically infected B6-CD45.1 mice could influence disease progression in uninfected MOG_(p35–55)_-immunized C57BL/6-CD45.2 recipients. Mice were immunized on day 0 with MOG_(35–55)_ in CFA and received either sterile PBS, 4×10^6^ CD4^+^ or 4×10^6^ CD4^−^ cells from *H. polygyrus*-infected donors on day 1. Pertussis toxin was given to all mice on days 0 and 2 (Fig. 5A). Progression of disease was monitored daily and assessed using a clinical score based on tail, hind limb and front limb paralysis as described in *Materials and methods*.

In two replicate experiments, CD4^+^ cells from infected donors were able to delay the onset of EAE and to reduce the severity of clinical signs observed in the first 2 wk (Fig. 5B). Beyond this time, in the recovery phase, the effect of CD4^+^ cell transfer was not reproducibly significant. However, MOG_(p35–55)_-immunized mice receiving CD4^−^ cells from infected donors consistently and significantly had a markedly lower clinical score, with a lower incidence and maximal clinical score throughout the time course observed, with an increased recovery rate in both experiments (Fig. 5C). The transfer of either CD4^+^ or CD4^−^ cells from *H. polygyrus-*infected mice did not alter the balance of Th1/Th2 responsiveness, as judged by unchanged MOG-specific recall splenocyte IFN-γ and IL-4 responses in recipient mice (data not shown).

### CD4^+^ and CD4^−^ cells from infected donors show similar long-term survival following transfer

In view of the more extended protection against EAE accorded by CD4^−^ cells, we compared the trafficking and survival of donor (CD45.1) cells in recipient mice 34 days after i.v. transfer of chronically infected MLNC. We have established that transferred B cells disseminate to many lymphoid tissues, but do not enter the CNS even under conditions of EAE (S M Anderton, unpublished). We therefore compared the numbers of donor genotype CD4^+^CD45.1^+^ (Fig. 5D) and CD4^−^CD45.1^+^ (Fig. 5E) cells recovered from the spleen and LN draining the site of immunization almost 5 wk after transfer. These data indicate that both cell types enjoy similar survival, and most likely proliferate, within the adoptive host.

### CD4^+^ cells from infected WT and IL-10^−/−^ mice also reduce the severity of EAE

**Figure 6:**
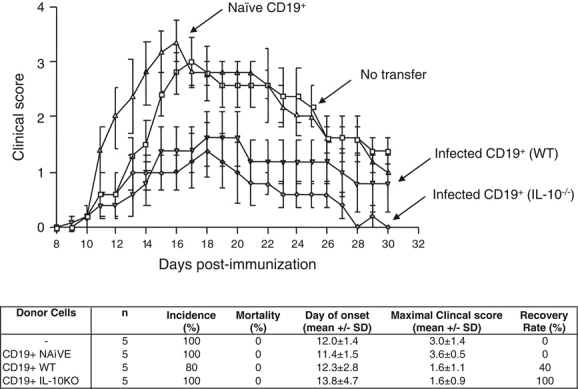
CD19^+^ cells from infected donors significantly reduce the severity of EAE in an IL-10-independent manner. Experiments were performed as detailed in Fig. 5A except that CD19^+^ cells from naïve, WT-infected and IL-10^−/−^-infected mice were transferred. Data are means±SE from five individual mice *per* group, with statistical significance assessed by the Mann–Whitney test (WT, *p*<0.004; IL-10^−/−^, *p*<0.0003). Summary data are shown in the lower panel.

To test the ability of helminth-induced B cells to modulate EAE, we adoptively transferred CD19^+^ cells from naïve, chronically infected WT and IL-10^−/−^ donors, into the tail vein of MOG_(35–55)_-immunized recipients. MOG_(35–55)_-immunized mice receiving CD19^+^ cells from infected donors, irrespective of IL-10 sufficiency, were significantly protected from EAE (Fig. 6). The onset of clinical signs was delayed with the maximal clinical score substantially reduced following the introduction of B cells from infected donors. The complete recovery was increased by 40 and 100%, at 30 days post-immunization, following the adoptive transfer of B cells from infected WT or IL-10^−/−^ donors, respectively.

### CD4^+^ B cells from infected mice are phenotypically similar to B2 cells and express high levels of CD23

The murine B-cell compartment is heterogeneous and can be separated into distinct subsets using a number of parameters including maturation stage, surface phenotype, anatomical localization and state of activation. CD5 and B220 have commonly been used to distinguish B1 and B2 cells, respectively [48] and CD23, the intermediate affinity IgE FcR, used to identify B-cell activation [49].

**Figure 7:**
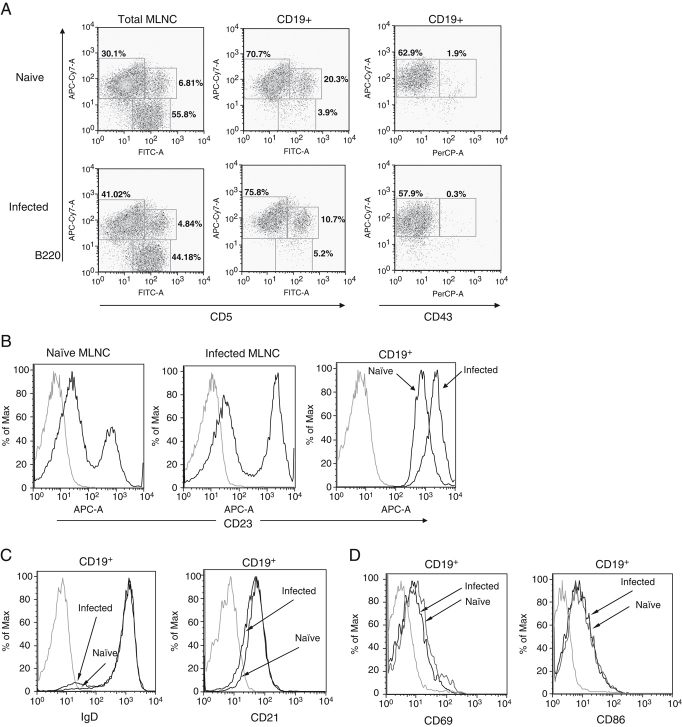
CD19^+^ cells from infected donors comprise B2 cells expressing uniformly high levels of CD23. (A) B220 co-staining with CD5 or CD43 markers for B1 cells in total MLNC and CD19^+^ purified cells from naïve C57BL/6 mice and mice infected for 28 days with *H. polygyrus*. Percentages indicate for each gate represent the mean value for infected MLNC from six individual mice, and two determinations from pooled naïve MLNC each from three individual mice. (B) Surface expression of CD23 on MLNC from naïve (left panel) and day 28-infected (middle panel) mice. CD23 expression in CD19^+^ B cells from naïve and day 28-infected mice is presented in the right-hand panel. Data are representative of four independent experiments. (C) Expression of IgD and CD21 by MLN CD19^+^ B cells isolated from naïve and day 28-infected mice. The proportion of CD19^+^ IgD-negative B cells rises from 6.67±0.79% in naïve MLNC to 12.18±0.71% in infected MLNC. Data are representative of four independent experiments. (D) Expression of CD69 and CD86 by MLN CD19^+^ B cells from naïve and day 28-infected mice. Data are representative of two independent experiments. Grey histogram represents the isotype control.

**Figure 8:**
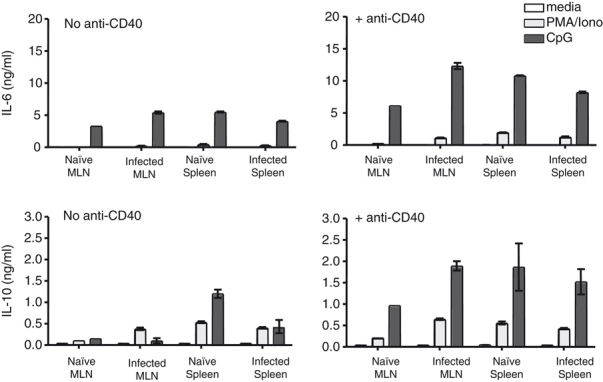
CD19^+^ cells from the MLN, but not the spleen, of helminth-infected mice produce greater IL-6 and IL-10 following TLR9 or polyclonal stimulation. IL-6 and IL-10 production by flow cytometry sorted MLN and spleen CD19^+^ B cells isolated from naïve and *H. polygyrus*-infected mice and stimulated *in vitro* with PMA-ionomycin or the TLR9 ligand CpG, in the absence (left hand panels) or presence (right hand panels) of agonist anti-CD40 antibody. Means and SEM of data from three replicate cultures are presented. Data are representative of two independent experiments.

To characterise the CD19^+^ B cells from the MLN of chronically infected mice, unseparated MLN and CD19-purified B cells were recovered and stained with CD19, CD5 and B220 to differentiate between B1 (CD5^int^ B220^int^) and B2 (CD5^low^B220^hi^) B-cell populations. In addition we stained for CD43, a marker known to be expressed by splenic B1 cells [50]. Using either unseparated MLN or CD19^+^ purified cells, the proportion of B1 cells in the MLN diminished following 28 days of infection with *H. polygyrus*, with very few CD43^+^ cells remaining (Fig. 7A). CD19^+^ B cells from infected mice, moreover, showed uniformly elevated levels of CD23, the low affinity FcɛR (Fig. 8B), with MFI of infected cells approximately twofold higher than cells from uninfected mice (1805±130 *versus* 854±19, *p*=0.0073). Infected MLNC contained a higher proportion of IgD-negative B cells, although little change was observed in CD21 expression (Fig. 8C). Few CD1d-positive B cells were observed in either naïve or infected MLNC populations (data not shown).

### CD4^+^ B cells from naïve and infected mice show similar levels of activation

To assess whether infection resulted in a generalised activation of mucosal-associated B cells, we also measured expression of CD69 and CD86, two surface markers up-regulated on activated B cells. Neither showed increased levels in B cells from infected mice, and indeed a small downshift in CD69 expression was evident in the population exposed to infection (Fig. 7D). Responsiveness to non-specific activation (PMA with ionomycin) or TLR ligation (with CpG) was also assessed, in terms of IL-6 and IL-10 cytokine production. In general, whether testing MLN or splenic populations, B cells from infected mice showed higher responses, particularly when co-stimulated with anti-CD40 antibody. However, the incremental effect was quite modest and there was no clear qualitative difference between the B-cell sets from naïve or infected mice (Fig. 8).

## Discussion

Helminth parasites are strongly associated with polarized Th2 responses [51–54], and have evolved a spectrum of anti-inflammatory mechanisms that limit immunopathology during chronic infection [31, 55]. Among these suppressive pathways, the role of CD4^+^CD25^+^ Treg has most clearly been substantiated [23, 25, 26, 56]. However, during infection alternatively activated macrophages, with the ability to dampen T-cell responses [33, 34, 57], along with dendritic cells providing negative signals to T cells [58], collectively suggest that a broader-based regulatory environment takes shape during helminth infections. Several studies in a range of systems have identified B-cell populations with significant regulatory potential [35, 38, 47, 59, 60]. Recent reports demonstrating regulatory activity of B cells in murine helminth infections [40, 43] have been followed by a report associating CD19^+^ B cells in helminth-infected MS patients with limited disease progression [44].

We accordingly tested whether B cells with regulatory capacity are generated by a chronic helminth infection, extending our earlier findings in the C57BL/6 strain, that both CD4^+^CD25^+^ and CD4^−^ subsets from *H. polygyrus*-infected mice are able to down-modulate allergic inflammation [23]. We found that, within the MLNC of chronically-infected mice, the CD19^+^ B-cell population exerts a suppressive effect on airway allergy, and that suppression is mediated in an IL-10-independent manner. Importantly, the inhibition of allergy cannot be attributed to a shift towards Th1 responsiveness, as it is well-established that *H. polygyrus* drives a strong Th2 response [23–25], and in our experiments no diminution in IL-4 production or IgE levels was observed. Furthermore, the same CD19^+^ subset is able to down-regulate inflammatory pathology in the Th1/Th17-mediated autoimmune model of EAE, also in the absence of IL-10 capability. Antibody levels in recipient animals were not significantly altered by B-cell transfer, suggesting that direct interactions with host cells may be necessary for suppression. Phenotypically, CD19^+^ B cells from infected donors are predominantly follicular-type B2 cells (B220^+^CD5^−^CD43^−^), and *in vivo* these cells are able to migrate to inflammatory sites and local draining LN following adoptive transfer.

Evidence is now building for an important role of B cells in regulation during helminth infections, acting to cap the magnitude of inflammatory responses. Earlier work had shown that *Schistosoma mansoni* infection of either μMT mice [61, 62] or JHD mice [63], both of which are deficient in B cells, leads to increased fibrosis, failure to down-modulate egg-induced granulomas in the liver (a feature observed in B-cell-competent mice) and a polarized Th1 response. These studies suggest that B cells assist in restraining potentially pathogenic responses, a role also ascribed to Treg cells in *S. mansoni* infection [64, 65]. More recently, B cells taken from *S. mansoni*-infected donors were shown to confer protection from Pen V-induced anaphylaxis and airway allergy [43], in an IL-10-dependent manner [40], while IL-10-secreting B cells were found to reduce antigen presentation and T-cell-driven inflammation during filarial helminth infections [41].

B cells operate interactively across the whole spectrum of immune system components [66], resulting in both direct and indirect influences on the T-cell compartment. Functional B-cell regulation of immunopathology has been established in EAE [47, 67, 68], chronic intestinal inflammation [35] and systemic lupus erythematosis [69, 70]. As antigen-presenting cells, B cells may initiate [71] or tolerise [72] the T-cell response. Furthermore, B cells can modify dendritic cell-orchestrated T-cell responses [73], indirectly interfering with T-cell activation and effector function. Most recently, and in the context of *H. polygyrus* infection, B cells have been shown to be major sources of key cytokines, including IL-2, IL-4 and TNF-α [74, 75]. In addition, of course, B cells are responsible for the production of antibodies, which are known to contribute towards immunity to *H. polygyrus* in mice [76]. It is interesting to note that we detected regulatory activity in B cells from C57BL/6 mice, which are fully susceptible to *H. polygyrus* and are slow to mount an antibody response, rather than from the more rapidly responding BALB/c strain, which develops faster immunity to the parasite.

In contrast to the above studies, we found that IL-10 is dispensable for *H. polygyrus*-induced B-cell protection from both airway allergy and EAE. Thus by both surface markers (CD5^−^, CD23^hi^) and by IL-10 independence, these helminth-induced Breg are dissimilar to the “B10” IL-10-producing regulatory B cells of the B1 or marginal zone type [37, 68]. We cannot exclude a role for host IL-10 mediating protection, a scenario observed with the adoptive transfer of OVA-specific Treg in a similar allergy model [77], or more closely related to these studies, adoptive transfer of apoptotic cell-exposed B cells licensing the generation of host IL-10-secreting T cells [59]. Varying dependence on IL-10 from helminth-induced B cells may reflect the specific target organs or tissues studied in the different systems. Moreover, immunological compensation may occur in CD19^+^ cells from *H. polygyrus*-infected IL-10^−/−^ donors, amplifying other mediators, such as TGF-β, as seen in the CTLA-4-deficient setting [78]. Notably, TGF-β expression is elevated in both CD4^+^ and CD4^−^ MLNC during *H. polygyrus* infection [23], and TGF-β signalling plays an important role in the T-cell response to this parasite [79]. As TGF-β has previously been associated with B-cell-regulatory function [36, 80], and is responsible for the ability of B cells to induce functional, Foxp3-expressing Treg cells [60, 81], the involvement of this mediator in helminth-induced B-cell suppression of pathology remains an attractive possibility.

A further factor in the suppression may be the uniformly high expression of CD23 on B cells from chronically infected mice. CD23 is the low affinity IgE receptor, expression of which is known to be amplified in gastro-intestinal nematode infection [82] and is positively regulated by levels of IL-4, IL-13 and IgE [83], all of which are elevated in *H. polygyrus* infection [53]. Furthermore, CD23 itself can have inhibitory effects on allergen-induced airway inflammation, with transgenic over-expression of CD23 limiting airway eosinophilia and airway hyper-responsiveness [84] and augmented airway responses in CD23^−/−^ mice [85, 86].

An alternative hypothesis is that B-cell down-regulation acts in a cell contact-dependent manner. FasL-expressing B cells, inducing apoptosis of antigen-specific T cells, have been reported to down-regulate airway inflammation [87], and significantly during *S. mansoni* infection this pathway of inducing T-cell apoptosis is enhanced [88]. Together, these observations provide an additional model of helminth-derived B-cell suppression of T-cell responses that is likely to be relevant to the study presented here.

In conclusion, we have identified CD19^+^CD23^hi^ B2 B cells, in addition to CD4^+^CD25^+^ Treg cells [23], as important components of the adaptive response to *H. polygyrus* infection, which have the capacity to down-modulate inflammatory reactions. Indeed, B-cell recruitment of Treg cells [89] might form a collaborative Treg–B-cell interaction, which continues throughout the chronic phase of infection. By including B cells within the interplay of immune mechanisms that develop during helminth infections, we can significantly broaden our understanding of the regulatory environment that forms in chronic infections [90], with important implications for the prospective exploitation of helminth products to combat major immune-dependent inflammatory disorders.

## Materials and methods

### Animals

Female BALB/c, C57BL/6 and congenic CD45.1-allelic C57BL/6 mice (B6-CD45.1), 6–8 wk old, were housed in individually ventilated cages). A minimum of five mice *per* group were used in each experiment. IL-10-deficient mice were on the C57BL/6 background [91]. All experimental animals were conducted following institutional ethical review and with authorisation of UK Home Office licence.

### Parasites

Mice were infected with 200 *Heligmosomoides polygyrus bakeri* infective L3 larvae using a gavage tube. After 28 days of infection, the chronicity of infection was confirmed with eggs recoverable from faecal pellets and gravid worms in the intestinal tract.

### Antigens, allergens and antibodies

For *H. polygyrus* antigen, adult worms were homogenised in PBS, followed by centrifugation (13 000 *g* for 10 min). Supernatant fractions were filtered through a 0.2 μm membrane (Millipore). Antigen concentrations were determined by the Coomassie Plus protein assay (Pierce). House dust mite allergen, *Der* p 1, was affinity-purified from spent mite medium using the monoclonal antibody 4C1 (INDOOR Biotechnologies, UK), according to published procedures [92]. Grade V Ovalbumin (A5503) was purchased from Sigma, UK.

### Allergen-induced airway inflammation

Mice were immunized by i.p. inoculation with 10 μg Der p 1 (C57BL/6) adsorbed to 9% potassium alum (Sigma A7167), and boosted again with 10 μg Der p 1 in alum i.p. 14 days later. On days 28 and 31, mice were anaesthetised with 20 μL/g body weight i.p. of a tribromoethanol anaesthetic, Avertin. Mice were then given two airway challenges with 10 μg Der p 1 in PBS by the intratracheal route. Mice were killed 24 h after the final airway challenge to assess airway inflammation. For histopathological analyses, formalin (4% paraformaldehyde in PBS) fixed lungs were processed and embedded in paraffin for sectioning. Hematoxylin and eosin stains (Sigma, 03972) were used for analysis of airway inflammation and pathological changes.

### Induction and assessment of EAE

EAE was induced by immunization with 100 μg of MOG_(p35–55)_ peptide emulsified in CFA containing 50 μg of heat-killed *Mycobacterium tuberculosis* H37RA (Sigma, Poole, Dorset, UK). The emulsion was administered as two 50-μL subcutaneous injections, one into each hind leg. Mice also received 200 ng of pertussis toxin (Speywood Pharmaceuticals, Maidenhead, UK) intraperitoneally in 0.5 mL of PBS on the same day and 2 days later. Clinical signs of EAE were assessed daily in a blinded fashion using a 0–6 scoring system (0, no signs; 1, flaccid tail; 2, impaired righting reflex and/or gait; 3, partial hind limb paralysis; 4, total hind limb paralysis; 5, hind limb paralysis with partial fore limb paralysis; 6, moribund or dead). Recovery rates are referred to as the percentage of mice with no observable clinical signs at day 30. Differences in total disease burdens between groups were analyzed with the Mann–Whitney *U*-test.

### BALF and differential cell counts

Twenty-four hours after the final challenge, mice were terminally anaesthetised with ketamine (Vetalar V, Pharmacia & Upjohn) and Xylazine (Rompun 2%, BAYER). The trachea was cannulated, and internal airspaces lavaged with an initial 500 μL of sterile PBS, followed by two 350 μL washes. Fluids were centrifuged at 1200 *g*, and pellets recovered for cellular analysis. The supernatants of the initial 500 μL of BALF were stored at −80°C for biochemical analyses. Cytospins were prepared by spinning 5×10^5^ cells onto poly-(l-lysine) coated slides (BDH) followed by Diff Quick® (Boehringer, UK) staining. Differential cell counts were performed at 100× magnification; a minimum of 200 cells were counted for each slide.

### Quantification of allergen-specific antibodies and total IgE

Allergen-specific responses were determined by ELISA. Multisorp (NUNC) plates were coated with 4 μg/mL Der p 1 diluted in 0.06 M carbonate buffer, and incubated overnight at 4°C. Plates were blocked with 5% BSA (Fraction V, Gibco) for 2 h at 37°C. Serum dilutions were added to plates in TBS, 0.05% Tween (TBS-T) and incubated overnight at 4°C. HRP-conjugated goat anti-mouse IgG1 (1070–05) and anti-IgG2a (1080–05) (both from Southern Biotech) and ABTS peroxidase substrate (50–62–00, KPL) were used to detect allergen-specific IgG isotypes. Prior to allergen-specific IgE assays, IgG was removed from sera by overnight incubation with protein-G sepharose beads; biotinylated anti-mouse IgE (Clone R35 118, BD Pharmingen), ExtrAvidin-Alkaline phosphatase conjugate (Sigma, E-2636) and pNPP Substrate (Sigma) were then used. Total IgE was measured with anti-mouse IgE capture antibody (clone R35–72, 553413, BD Pharmingen) and biotinylated anti-mouse IgE detection antibody (clone R35–118, 553419), using a monoclonal IgE standard curve (clone 27–74, 553481).

### Cytokine and chemokine (eotaxin) measurements

Cytokines were measured by ELISA according to suppliers' guidelines as previously described [24]. Capture antibodies used were: for IL-5, TRFK5, 2 μg/mL; IL-6, MP5-20F3, 2 μg/mL; IL-10, JES5-2A5, 4 μg/mL; eotaxin AF420NA, 0.4 μg/mL. Biotinylated detection antibodies were purchased from Pharmingen for IL-5 (TRFK4, 2 μg/mL), IL-6 (MP532C11, 0.5 μg/mL) IL-10 (SXC-1, 2 μg/mL) or R&D systems (Eotaxin, mAb BAF420, 0.4 μg/mL).

### CD4^+^, CD4^−^ and CD19^+^ isolation and adoptive transfer

MLN were removed from mice infected 28 days earlier with 200 *H. polygyrus* larvae. Single-cell suspensions in RPMI-0.5% normal mouse serum were made using a cell strainer. For CD4^+^ cell purification, cell suspensions were incubated with CD4 (L3T4) microbeads (Miltenyi Biotech, Bisley UK 130–049–201) and separated on two MACS LS separation columns (130–042–401) to enhance purity after filtering with pre-separation filters (130–041–407); 4×10^6^ CD4^+^ or CD4^−^ cells were injected i.v. into recipient mice. For CD19^+^ cell purification, MLNC were first incubated with CD19 microbeads (Miltenyi Biotech 130–052/201) and separated on two MACS LS separation columns as above. Uninfected, allergen-sensitized mice received cells from infected donors 7 days before the first airway challenge. MOG_(p35–55)_-immunized mice received cells from infected donors 1 day after MOG_(p35–55)_ immunization.

### *In vitro* B-cell culture

Following a previously published protocol [93], single cell suspensions from the MLN and spleens of naïve and infected C57/BL6 mice, were prepared in complete Iscove's modified Dulbecco's medium (cIMDM+10%FCS+1% penicillin/streptomycin+1% l-glutamine). Following red blood cell lysis, cell preparations were labelled with anti-CD19 microbeads and separated by positive selection on a magnetic column, according to the manufacturer's instructions (Miltenyi Biotech). Purified B cells were cultured in triplicate at 5×10^6^ cells/mL in cIMDM in the presence or absence of anti-CD40 (clone FGK-45, 10 mg/mL), and either CpG (25 mg/mL) or PMA and ionomycin (Sigma UK) (50 ng/mL and 1 mg/mL respectively). Supernatants were collected after 5 days and stored at −80^o^C until used for cytokine analysis.

### Flow cytometry

Cells were stained with antibodies diluted in PBS with 0.5% BSA (Sigma-Aldrich), 0.05% sodium azide (Sigma-Aldrich) for 20 min at 4°C. For detection of CD4^+^CD45.1^+^ donor cells, monoclonal rat anti-mouse CD4 (L3T4, clone RM4-5, Isotype Rat IgG2a) and rat anti mouse-CD45.1 (Ly5.1, Clone A20, Isotype Mouse IgG2a) were used. The expression of surface markers was analysed on FACSCalibur or LSRII flow cytometers using FlowJo software (Tree Star). All fluorochrome-labelled antibodies were obtained from BD Pharmingen, unless otherwise stated. For detection of B–cell-associated markers, the monoclonal rat IgG2a antibodies were used to the following antigens: B220 (clone RA3 6B2), CD5 (Clone 53-7.3), CD19 (Clone 1D3, E-Bioscience), CD21 (Clone 7E9, BioLegend), CD23 (Clone B3B4, BioLegend), CD43 (Clone 1B11, BioLegend), CD69 (H1.2F3), CD86 (GL1) and IgD (11–26c.2a).

### Splenocyte and LN re-stimulation

Spleens or LN were immediately removed from recently killed mice, sieved into single-cell suspensions in RPMI-0.5% normal mouse serum using a cell strainer. Cells were incubated in quadruplicate at 1×10^6^ cells/well of a round-bottomed 96-well plate in a total of 200 μL at 37°C. Supernatants were collected, after 54 h of culture, and stored at −80°C for cytokine analysis.

### Statistical analysis

The Mann–Whitney test was used for all statistical comparisons, unless otherwise stated; *p* values <0.05 were considered significant.

## Abbreviations

MLNC: MLN cells
MOG: myelin oligodendrocyte glycoprotein
