# Supplementary material for: Helminth-induced CD19+CD23hi B cells modulate experimental allergic and autoimmune inflammation
Source: Eur J Immunol. 2010 Mar 19;40(6):1682–96. doi: 10.1002/eji.200939721 (PMC3179601; doi:10.1002/eji.200939721)
Supplement: Supplementary file 1 [file eji0040-1682-SD1.pdf]

// European Journal of Immunology

European Journal of Immunology

Supporting Information

for

DOI 10.1002/eji.200939721

Helminth-induced CD19^{+}CD23^{hi} B cells modulate experimental allergic and autoimmune inflammation

Mark S. Wilson, Matthew D. Taylor, Mary T. O'Gorman, Adam Balic,
Tom A. Barr, Kara Filbey, Stephen M. Anderton and Rick M. Maizels

© 2010 WILEY-VCH Verlag GmbH & Co. KGaA, Weinheim

Wilson *et al.* Suppl. Figure 1

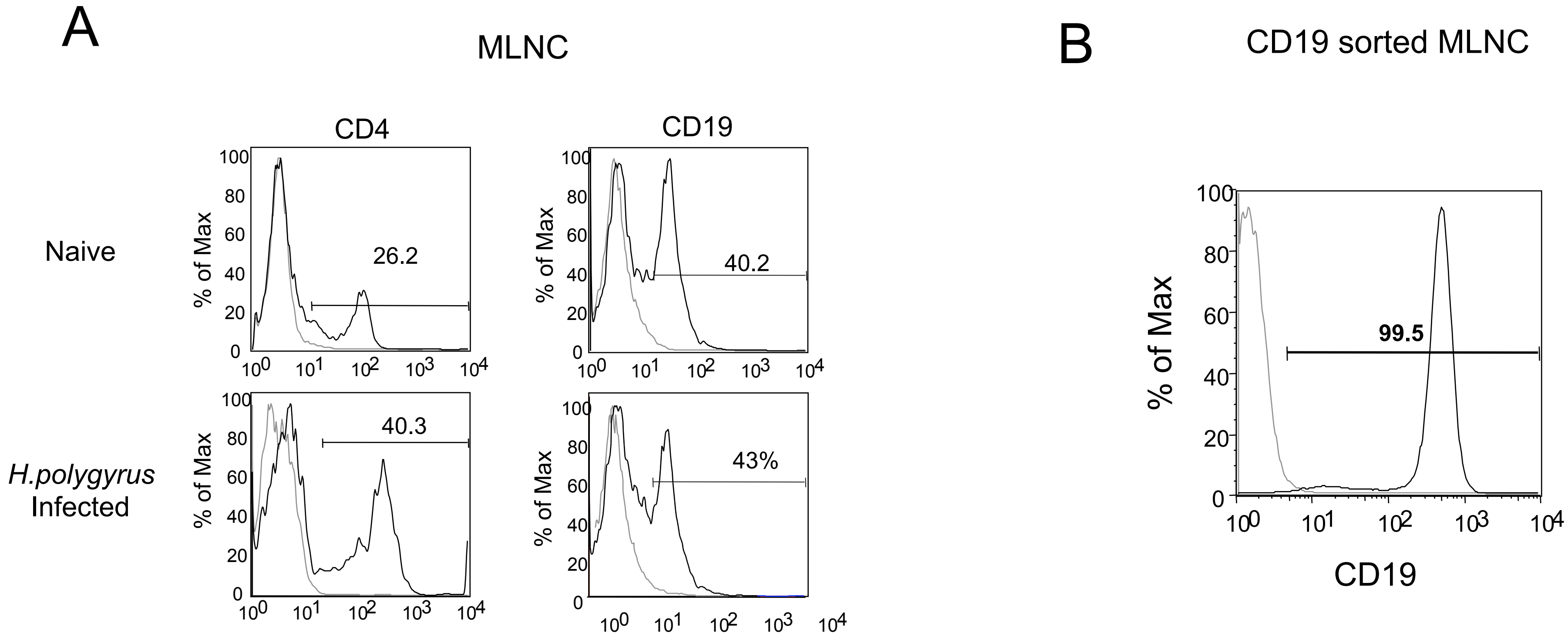

Supplementary Figure 1.

Proportion and purity of CD19+ cells isolated form the MLN of helminth-infected mice.

A. CD4 and CD19 expression in MLNC from naïve and day 28-infected C57BL/6 mice.

B. $CD19^{+}$ MACS purification from day 28-infected C57BL/6 mice.
